# Prevalence and risk factors for *Brucella* seropositivity among sheep and goats in a peri-urban region of Tajikistan

**DOI:** 10.1007/s11250-015-0992-3

**Published:** 2016-01-15

**Authors:** Elisabeth Lindahl Rajala, Cecilia Grahn, Isabel Ljung, Nosirjon Sattorov, Sofia Boqvist, Ulf Magnusson

**Affiliations:** Division of Reproduction, Department of Clinical Sciences, Swedish University of Agricultural Sciences, P.O. Box 7054, SE-750 07 Uppsala, Sweden; Tajik Agrarian University, P.O. 734003, Dushanbe, Tajikistan; Division of Food Safety and Bacteriology, Department of Biomedical Sciences and Veterinary Public Health, Swedish University of Agricultural Sciences, P.O. Box 7028, SE-750 07 Uppsala, Sweden

**Keywords:** Brucellosis, Tajikistan, Small ruminants, ELISA

## Abstract

This cross-sectional study aimed to estimate the seroprevalence of *Brucella* infection among sheep and goats on small-scale farms in a peri-urban area of Tajikistan and identify factors associated with seropositivity. The study population was 667 female sheep and goats >6 months of age from 21 villages in four districts surrounding the capital city, Dushanbe. Individual blood samples were collected during October and November 2012 and analysed with indirect enzyme linked immunosorbent assay (ELISA). Positive samples were confirmed with competitive ELISA. To identify factors associated with seropositivity at an individual level, a generalised linear mixed model was applied to account for clustering of individuals within villages and districts. The true individual seroprevalence was 6.7 % and ranged from 1.0 to 15.6 % between the four districts. Fourteen villages had at least one seropositive sheep or goat, resulting in apparent prevalence of 67 % at village level. The seroprevalence at individual level was significantly lower in Rudaki district (odds ratio (OR) = 0.1; 95 % confidence interval (CI) 0.03–0.4) and Varzob district (OR = 0.3; 95 % CI 0.09–0.8) than in Vahdat district. Sheep were more likely than goats to be seropositive (OR = 2.7; 95 % CI 1.3–5.5). Increasing age was significantly associated with seropositivity (OR = 1.4; 95 % CI 1.2–1.6). These results indicate high prevalence of *Brucella* infection among sheep and goats in the peri-urban area of the capital city in Tajikistan. Given the dense human population in such areas, this could constitute a threat to public health, besides causing significant production losses.

## Introduction

Brucellosis is one of the most common and economically important zoonoses globally (McDermott et al. 2013). Central Asia, the Middle East and adjacent subtropical geographies are among those with the highest incidence of brucellosis among humans and livestock worldwide (Pappas 2010). There is a reason to believe that the burden caused by brucellosis in low-income countries in Asia and Africa is large (McDermott et al. 2013).

In humans, the disease can cause chronic infection if not treated adequately, with osteoarticular manifestation being a common complication (Dean et al. 2012). Brucellosis in livestock mainly affects the reproductive organs and causes abortion, reduced fertility and decreased milk production. The disease can have serious negative impacts for people living in low-income countries, like loss of work or income due to illness and disability, and can cause significant economic losses in countries depending on their livestock sector (McDermott et al. 2013). The different *Brucella* spp. infecting livestock are *Brucella melitensis* (mainly infecting sheep and goats), *Brucella abortus* (mainly infecting cattle) and *Brucella suis* (mainly infecting swine), all of which have zoonotic potential (Godfroid et al. 2011). *B. melitensis* is the most common cause of human brucellosis worldwide (Blasco and Molina-Flores 2011).

Following the collapse of the Soviet Union, small-scale farming in Tajikistan increased substantially and is a common practice today in rural areas and in urban and peri-urban areas. The animal health situation in the country is poor, prompting the UN Food and Agriculture Organization (FAO) to initiate a brucellosis vaccination programme among sheep and goats in high prevalence areas in 2004 (Ward et al. 2012). The programme did not include the districts surrounding the capital, Dushanbe. In a follow-up survey in 2009, the overall *Brucella* seroprevalence among sheep and goats was 1.8 % in well-vaccinated districts and 4.2 % in non-vaccinated districts (Ward et al. 2012).

The objectives of the present study were to estimate the seroprevalence of *Brucella* infection among sheep and goats in peri-urban small-scale farming in Tajikistan and to identify factors associated with seropositivity among sheep and goats.

## Materials and methods

### Study area and study population

This cross-sectional study was performed in peri-urban areas within a 30-km radius of central Dushanbe. The livestock production is categorised as a rangeland-based arid/semi-arid or tropical highland system (Robinson et al. 2011).

**Fig. 1:**
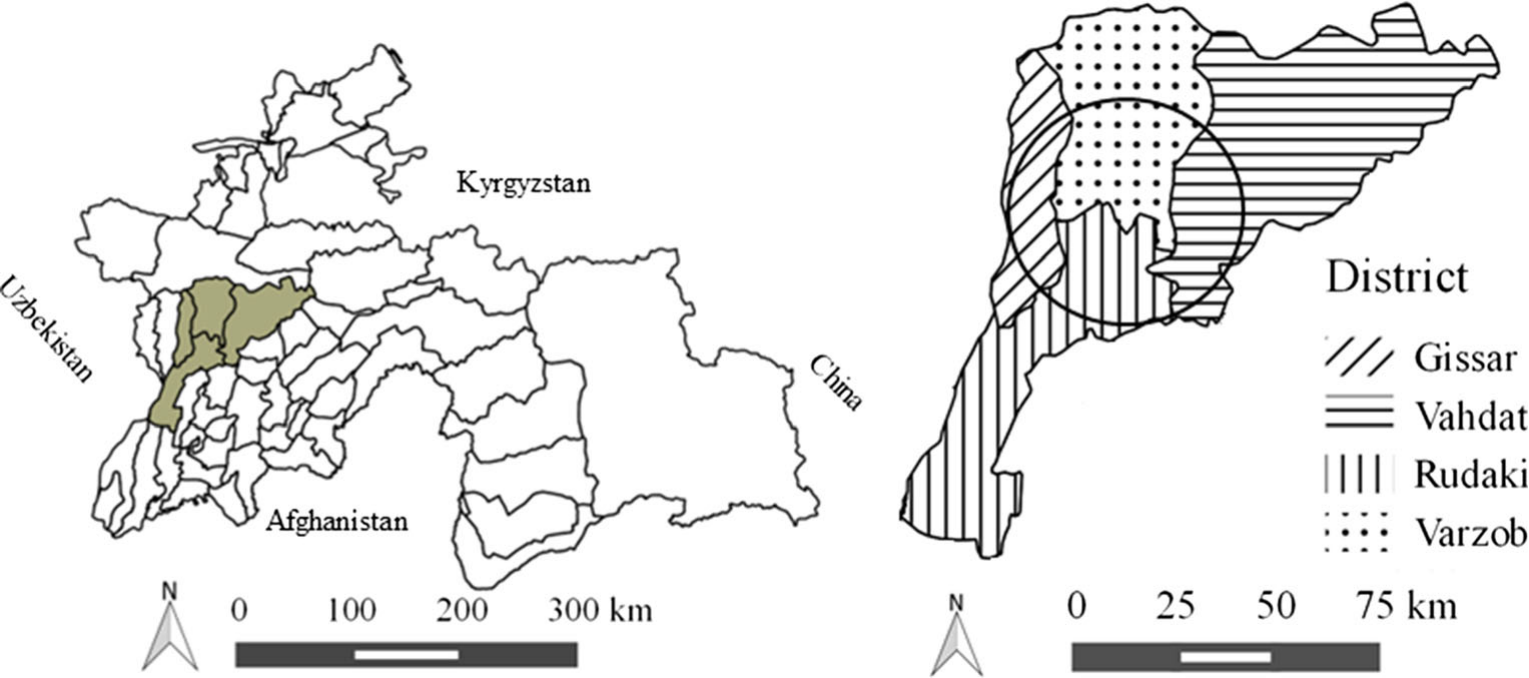
*Left*: General map of Tajikistan showing the four districts included in the study. *Right*: Detailed map of the four districts, with the approximate outer border of the study area represented by a *black circle* (Quantum GIS 2.4.0, Chugiak)

The study was restricted to peri-urban areas because few sheep and goats are kept within the city due to limited access to natural rangelands. Dushanbe is populated by approximately 750 000 people (UN 2015), and 300 000 sheep and goats are kept in the districts surrounding the capital (state veterinary service’s official records). The villages included in the study are located in four districts neighbouring Dushanbe: Varzob, Gissar, Rudaki and Vahdat (Fig. 1). The area is dominated by small-scale farming, most commonly with <20 sheep and goats and 1–3 cows per household. Sheep and goats are most commonly used for meat production and to a lesser extent for milk production. An average-sized village in the study area has approximately 100 households, and these peri-urban villages often have access to vast pastures where communal grazing is common between May and October. The study population was 667 female sheep and goats >6 months of age that had not been vaccinated against brucellosis (state veterinary service’s official records). All sheep included were of the fat-tailed Gissar breed, and all goats included were of the local Tajik breed.

### Study design

As many samples as possible were collected with a minimum of 385 individual blood samples, to estimate the seroprevalence of *Brucella* infection at an individual level with an expected prevalence of 50 %, a confidence level of at least 95 % and a desired absolute precision of at least 5 %. The samples were distributed over the four districts. The villages included had to be located with a radius of <30 km of central Dushanbe and be accessible by car. Another inclusion criterion was that the study team could receive assistance in the village during sampling. Information on the villages keeping sheep and goats was obtained from local official veterinarians. The aim was to visit 20 villages, one per day. The villages that met the inclusion criteria were listed for each district Varzob (north), Gissar (west), Rudaki (south) and Vahdat (east) in order to include villages spread around the city. Five or six villages were randomly selected from each district. In each village, the animals were sampled either within the household or on pasture. In all villages except five, at least 20 animals belonging to five different households were sampled. The selection of households within each village was performed on site and based on whether the householder was present and willing to participate in the study. A maximum of 10 individual animals that met the inclusion criteria were sampled per household. If a household had more than 10 sheep and goats, the selection was performed by the owner or the study team. If the animals were on pasture, the study team sampled the animals they managed to catch.

The samples were collected during October and November 2012. This period was chosen as the majority of the animals are kept in or close to the village at this time of year and were thus easy to sample.

### Collection of blood samples and epidemiological data

The blood samples were collected from the jugular vein with a Vacutainer into sterile tubes without additions (BD Vacutainer Systems, Plymouth, UK). They were kept cold during transport to the laboratory at Tajik Agrarian University in Dushanbe. The serum was removed after centrifugation and stored at −20 °C until analysis at the university. No sample was stored longer than 8 weeks.

A questionnaire was used to collect epidemiological data about each sampled animal. Either a household member or the person responsible for herding the animals on pasture was interviewed by the same person throughout the study. Data collected for each individual animal were as follows: age, species, pasture type, history of abortion/stillbirth and name of the district and village. Each animal was linked with the same number on the questionnaire and the blood sample. No data regarding the identity of the farmers or individual animals were collected.

### Serological analyses

The serological analyses were performed at Tajik Agrarian University using a commercial enzyme-linked immunosorbent assay (ELISA) (SVANOVA Biotech AB, Uppsala, Sweden) according to the manufacturer’s instructions. All samples were initially screened with indirect ELISA (I-ELISA) according to the World Organisation for Animal Health recommendations (OIE 2009). Positive samples were confirmed with competitive ELISA (C-ELISA). A sample was regarded as seropositive to *Brucella* when it tested positive in both I-ELISA and C-ELISA. The ELISAs used do not distinguish *B. abortus* from *B. melitensis*. In the I-ELISA, samples were considered positive if percentage positivity (PP) was ≥15. The PP was calculated as (mean OD (optical density)_sample_ / mean OD_positive control_) × 100.

In the C-ELISA, samples were considered positive if percentage inhibition (PI) was ≥30. The PI was calculated as 100 − ((mean OD_samples_ × 100) / (mean OD_conjugate control_)). For I-ELISA, according to the manufacturer, the sensitivity (Se) is 0.94 and the specificity (Sp) 1.0. A study specifically on goats has shown that for I-ELISA, Se is 0.96 and Sp is 1.0, while for C-ELISA, Se is 0.94 and Sp is 0.99 (Nielsen et al. 2005). The estimated Se for the test series is 0.88 and the Sp is 1.0.

All samples were run in duplicate, and test validation was performed with positive and negative controls according to the manufacturer’s instructions. The true prevalence at individual level was calculated according to Rogan and Gladen (1978) using the Se and Sp for the C-ELISA as follows:

$$ \mathrm{T}\mathrm{P}=\left(\mathrm{A}\mathrm{P}+\mathrm{S}\mathrm{p}-1\right)/\left(\mathrm{S}\mathrm{e}+\mathrm{S}\mathrm{p}-1\right) $$

where TP is true prevalence and AP is apparent prevalence.

### Statistical analyses

Data were entered in Excel (Microsoft) and statistical analyses performed in SAS version 9.3 (Cary, NC, USA). All villages practised communal grazing, and the village was considered to be the epidemiological unit. To account for clustering of individuals within villages and district, logistic regression analysis using a generalised linear mixed model was used. The analyses were carried out using the Glimmix procedure in SAS. All variables were categorical except for the continuous variable Age. The variables pasture type and history of abortion/stillbirth were excluded from the statistical analyses due to homogeneous answers. The other variables were screened with univariate analyses and entered in a multivariable model at individual level to investigate potential associations between seropositivity and the different factors. Manual backward elimination was used until all remaining variables showed a two-tailed *P* value <0.05. The model was investigated for interactions between all variables included in the final model. Confounding was investigated by adding potential eliminated variables in the final model. The variables in the final model were also excluded one by one to test whether any of the significant variables was a confounding factor. A variable was considered to be a confounder if it changed the coefficient of the significant variables by >25 %.

## Results

### Description of study population

None of the farmers refused to participate in the study. The initial dataset contained 871 individual blood samples from sheep and goats. The results from three I-ELISA plates and one C-ELISA plate had to be excluded due to problems with validation of the positive and negative controls. In total, 667 individual blood samples were included, from 260 sheep and 407 goats in 21 villages. On average, 32 samples were collected per village (range 4–55 animals). The median age of the sheep and goats was 3 years (min = 0.5, Q1 = 2, Q3 = 4, max = 15). One goat was reported to have a history of abortion/stillbirth. All villages in the study practised communal grazing. The descriptive results are summarised in Table 1.

**Table 1:** Descriptive results of *Brucella* seropositivity at individual level (*n* = 667), Tajikistan, 2012

Variable	Category	Number (%)	Seropositive number (%)
Species	Sheep	260 (39)	28 (11)
	Goat	407 (61)	20 (5)
District	Varzob	174 (26)	5 (3)
	Rudaki	156 (23)	3 (2)
	Gissar	156 (23)	12 (8)
	Vahdat	181 (27)	28 (15)

### *Brucella* seropositivity and associated factors at individual level

The true individual seroprevalence was 6.7 % and ranged from 1.0 to 15.6 % in the four districts. None of the animals below 1 year (*n* = 59) were seropositive. Fourteen villages had at least one seropositive sheep or goat, resulting in apparent prevalence at village level of 67 %. As all villages practised communal grazing and only one animal was reported to have had problems with abortion/stillbirth, these factors were not investigated further.

The result from the multivariable analysis revealed that sheep were significantly more likely to be seropositive than goats (*P* = 0.009) (Table 2). An increase in age was associated with a significant increase in seropositivity (*P* < 0.001). There was also a significant difference in seroprevalence between the districts. Sheep and goats in Rudaki (*P* = 0.003) and Varzob (*P* = 0.024) were less likely to be seropositive than sheep and goats in Vahdat. No interactions or confounding were found in the model.

**Table 2:** Relationship between associated factors and *Brucella* seropositivity at individual level (*n* = 667) using multivariable logistic regression analyses with village as random effect, Tajikistan, 2012

Variable	Category	*β*	*P*	OR (95 % CI)
Species	Sheep	1.0	0.009	2.7 (1.3–5.5)
	Goat			
District			0.008	
	Varzob	−1.3	0.024	0.3 (0.09–0.8)
	Gissar	−0.5	0.191	0.6 (0.3–1.3)
	Rudaki	−2.3	0.003	0.1 (0.03–0.4)
	Vahdat			Reference
Age (in years)	Continuous	0.3	<0.001	1.4 (1.2–1.6)

## Discussion

This study indicates that *Brucella* infection among sheep and goats in a peri-urban area of Tajikistan is more prevalent than has been reported from rural areas and that there are substantial differences between the four peri-urban districts investigated.

The seroprevalence in Vahdat district was significantly higher than that in Rudaki and Varzob districts. Sheep were more likely to be seropositive than goats, and increased age was positively associated with seropositivity.

The true individual seroprevalence was 6.7 %, with a significant difference (1.0–15.6 %) between the four districts. As none of the animals had been vaccinated against brucellosis, seropositivity was considered to be caused by natural exposure to infection. In a serosurvey performed in 2009, the seroprevalence was 4.2 % in rural districts bordering Dushanbe (Ward et al. 2012). The 50 % higher seroprevalence in peri-urban areas in the present study than the rural average (and more than threefold higher seroprevalence in one peri-urban district) is an animal and public health concern, given the higher human population density in the peri-urban areas. Notably, only one goat was reported to have a history of abortion/stillbirth. One explanation to this low number could be that the farmers fail to observe the abortions/stillbirth at pasture. Another reason could be that abortion due to *Brucella* infection is relatively uncommon in some areas (Corbel 2006). One reason for the high seroprevalence observed in Vahdat district could be that many villages from other districts use part of the district as main road for the movement of sheep and goats between summer and winter pastures. This increases the contact between animals from different villages and hence the risk of transmission of *Brucella* infection. Furthermore, there are three large animal markets in Vahdat district that could play an important role in transmitting disease. To reduce the risk of transmission of brucellosis between villages and districts, trade in animals should be restricted (Blasco and Molina-Flores 2011). A study in the neighbouring country of Kyrgyzstan showed slightly lower *Brucella* seroprevalence of 3.3 % in sheep and 2.5 % in goats (Bonfoh et al. 2012) than observed here and also showed an association between seroprevalence in humans and small ruminants.

Sheep were more likely to be seropositive than goats in the current study. However, other literature suggests that goats are more susceptible to *B. melitensis* infection than sheep (Quinn et al. 2011. There was no difference in seroprevalence between non-vaccinated sheep and goats in a previous study conducted in Tajikistan (Ward et al. 2012). Differences in susceptibility have been observed among sheep where the milking breeds seem to be most susceptible to *B. melitensis* (Corbel 2006). More research is required to allow firm conclusions to be drawn on whether sheep of fat-tailed Gissar breed are more susceptible to *Brucella* infection than goats.

An increase in age was associated with increased seropositivity, and none of the animals <12 months of age were seropositive. This corresponds with the biology of *Brucella* that younger animals are more resistant to infection than sexually mature animals and has also been observed in other studies (Boukary et al. 2013; Akbarian et al. 2015). Older animals have also had a longer time to be exposed to infection compared to younger animals.

As discussed previously, the data regarding abortion/stillbirth might not represent the true number of abortions in the study area considering the high seroprevalence in some districts. There may also have been a potential bias in the current study owing to difficulties in achieving perfect random selection of villages, as they had to meet certain inclusion criteria to allow collection of samples by the study team. However, it is difficult to find any reason why the inclusion criteria would affect the outcome of the study. Another potential bias was the difficulties in performing a random selection of individual animals. However, as the majority of the small ruminants in each household were included in the study, the risk of selection bias is estimated to be minor. In households with more than ten small ruminants, the potential selection bias is considered to have been mitigated by the fact that the selection was performed by different persons and not only the animal owner. We therefore consider the results presented in this study to provide a representative picture of the occurrence of *Brucella* infection and risk factors associated with seropositivity among sheep and goats in the study area.

The results indicate that *Brucella* infection is endemic, with a high prevalence, among sheep and goats in peri-urban areas surrounding the capital city of Tajikistan. Whether this higher seroprevalence in peri-urban areas compared with values reported for rural areas is a common pattern valid for other parts of the world remains unclear, as comparative data are scarce. Peri-urban areas are often highly populated, and if brucellosis is endemic among livestock in peri-urban areas, this could constitute a risk to public health. It is therefore important to include peri-urban areas when investigating the occurrence of *Brucella* infection among livestock and implementing control programmes.
